# Optimizing Membrane Composition With C15 and Choline: A Novel Hypothesis to Mitigate Cancer Risk

**DOI:** 10.7759/cureus.99954

**Published:** 2025-12-23

**Authors:** Joseph Mercola

**Affiliations:** 1 Research, Midwestern University Chicago College of Osteopathic Medicine, Downers Grove, USA

**Keywords:** c15:0, choline, chronic inflammation, linoleic acid, lipid peroxidation, membrane phospholipids, pentadecanoic acid

## Abstract

Cancer incidence has dramatically increased over the past centuries, paralleling dietary shifts in Western nations. This narrative review evaluates whether excess dietary linoleic acid (LA) drives tumor initiation through membrane-mediated oxidative stress and chronic inflammation, and whether pentadecanoic acid (C15:0) and choline can restore membrane resilience and attenuate cancer risk. Historical epidemiological data and mechanistic studies reveal that rising cancer incidence tracks population-level LA exposure, while C15:0 and choline consumption have declined. In cellular and animal models, LA oxidation products activate pro-inflammatory and anti-apoptotic pathways. Conversely, C15:0 resists peroxidation and preserves mitochondrial integrity, while choline normalizes membrane fluidity. A precision-nutrition strategy restricting LA while fortifying C15:0 and choline offers a biochemically coherent complement to conventional oncology. Prospective clinical trials are warranted to validate whether correcting this lipid imbalance can reduce cancer incidence.

## Introduction and background

**Figure 1:**
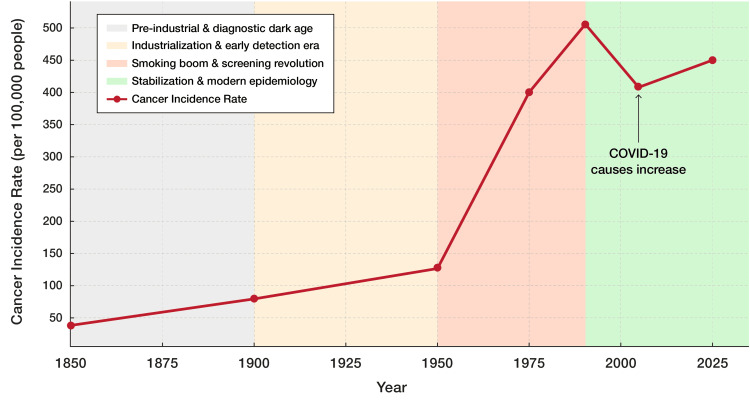
Cancer incidence rates in the United States over time (1850-2025) Historical trend of cancer incidence rates in the United States from 1850 to 2025, showing the rate per 100,000 people. Rates rose from approximately 50 in 1850 to 500 by 2025. The graph spans four eras: pre-industrial and diagnostic dark age (1850-1900), industrialization and early detection era (1900-1925), smoking boom and screening revolution (1925-2000), and stabilization and modern epidemiology (2000-2025). A temporary dip in 2020 reflects COVID-19-related disruptions in diagnosis. Image Credit: Joseph Mercola, DO

Cancer has evolved from a relatively rare affliction in the 19th-century United States (U.S.) to one of the nation’s leading health challenges by the 21st century (Figure 1) [1-4]. In the 1840s, Rudolf Virchow’s histological studies (microscopic tissue analysis) established that neoplasms (abnormal tissue growths), such as carcinomas, derive from pre-existing cells, refuting 19th-century spontaneous generation dogma and laying oncology’s cellular foundation [5]. By 1900, his framework was fully accepted in U.S. medical schools and hospitals, tightening diagnostic criteria and setting the stage for the steep, data-rich rise in recorded incidence that followed.

Initially, in the pre-industrial era, cancer incidence lingered at 60-80 cases per 100,000, masked by short lifespans averaging 35 years and limited diagnostics like microscopy [6]. By 1950, rates rose to 130-160 per 100,000 [7], driven by industrialization, the emergence of carcinogens such as asbestos, and increased life expectancy to 68 years, expanding the aging population at risk.

The smoking epidemic, peaking in the 1960s with 70% of men smoking [8], propelled lung cancer rates, pushing overall incidence to 400 per 100,000 by 1975. Incidence peaked at 505 per 100,000 in 1992 [9], fueled by prostate-specific antigen (PSA) screening [10]. However, a decline began around 1990 [11], likely due to reduced smoking following the Surgeon General’s warnings in the 1970s and 1980s, with a 20-30-year lag reflecting decreased lung cancer rates [12,13]. By 2020, incidence stabilized at 440-450 per 100,000 [14], despite a temporary dip to 404 per 100,000 due to COVID-19 disruptions [15], underscoring cancer’s persistent rise shaped by lifestyle, detection, and demographics. This upward trend underscores the need to investigate underlying factors contributing to cancer risk, including dietary shifts and environmental exposures.

Against this backdrop, dietary patterns, particularly the modern rise in linoleic acid (LA), rich seed oils, emerge as plausible, mechanistically supported contributors to contemporary cancer risk and therefore form the focus of this narrative review. First, this review examines how the omega-6 fatty acid LA integrates into membrane phospholipids and cardiolipin (CL), establishing its essential but potentially hazardous biochemical role. Second, it details the mechanisms by which LA-derived oxidation products activate inflammatory and anti-apoptotic pathways that favor tumor survival. Third, pentadecanoic acid (C15:0) and choline as membrane-stabilizing nutrients capable of counteracting LA-induced dysfunction are introduced. Finally, limitations and proposed future research directions are discussed to validate this precision-nutrition hypothesis.

Literature search and methodology

This narrative review synthesizes evidence from peer-reviewed literature identified through PubMed, Google Scholar, and Web of Science databases. Search terms included combinations of "linoleic acid", "membrane phospholipids", "lipid peroxidation", "cardiolipin", "pentadecanoic acid", "C15:0", "choline", "NF-κB", "cancer", "inflammation", and "oxidative stress". No date restrictions were applied to capture foundational historical studies alongside contemporary mechanistic and epidemiological research through early 2025. Historical cancer incidence data were derived from peer-reviewed epidemiological publications and National Cancer Institute reports. Dietary intake trends were sourced from the National Health and Nutrition Examination Survey (NHANES) datasets and published analyses of adipose tissue fatty acid composition. Studies were selected based on relevance to the central hypothesis linking membrane lipid composition to cancer risk through oxidative and inflammatory mechanisms. This review does not employ systematic methodology with predefined inclusion/exclusion criteria; rather, it synthesizes mechanistic, observational, and experimental evidence to generate a testable hypothesis for future investigation.

## Review

**Figure 2:**
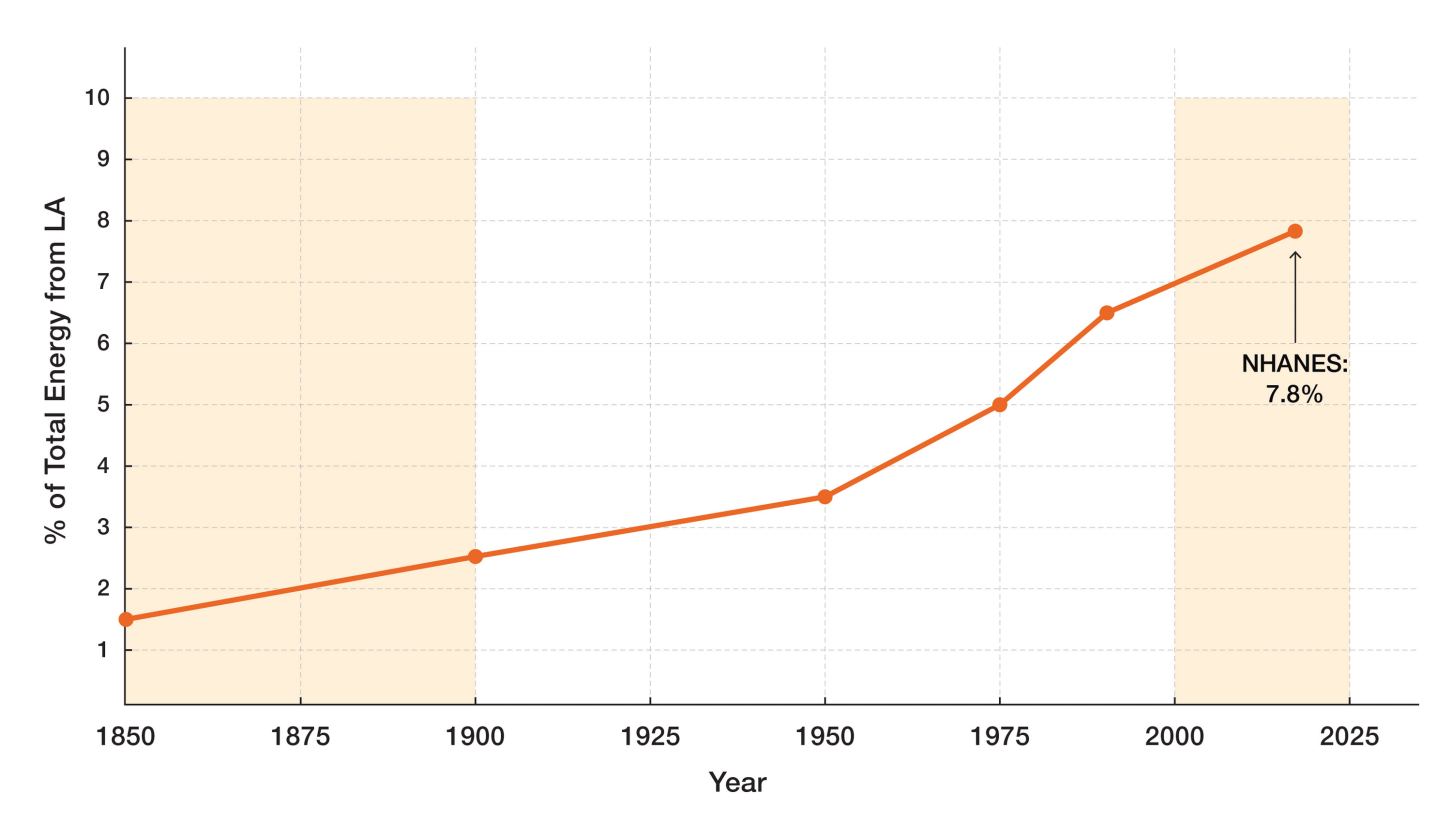
Linoleic acid intake as percentage of total energy (1850-2020) Estimated increase in linoleic acid (LA) intake as a percentage of total energy from 1850 to 2020, paralleling the rise in cancer incidence. The data illustrate the transition from ancestral diets (1-2% LA) to modern Western diets (7-10% LA), with National Health and Nutrition Examination Survey (NHANES) data confirming a current mean of approximately 7.8% [27]. Image Credit: Joseph Mercola, DO

Cancer, a multifactorial disease, arises from genetic predisposition, environmental carcinogens, and lifestyle factors [16], including sedentary behavior and dietary patterns [17,18]. Among these, chronic inflammation [19], exacerbated by the industrialization of the food supply [20,21], has emerged as a major cause of death. Historical data (Figure 2) and recent evidence implicate excess LA (18:2 n-6) as a potential contributor to rising cancer incidence [22,23]. In contemporary Western diets, LA constitutes 7-10% of total energy intake, a stark contrast to the 1-2% typical of ancestral Paleolithic diets [24]. Between 1959 and 2008, LA concentration in subcutaneous adipose tissue of the U.S. population surged by 136%, from 9.1% to 21.5% of total fatty acids [25], prompting scrutiny of its role in oncogenesis [26].

Cross-population data reinforce this trend. In the mid-20th century, isolated communities had LA compositions ranging from 5.5% to 10.6% of total serum fatty acids (Table 1) [28,29]. 

**Table 1:** Proportion of linoleic acid in subcutaneous adipose tissue or blood lipids by population (1950s-1960s vs. 2025) Percentage of linoleic acid as a proportion of total fatty acids in subcutaneous adipose tissue or blood lipids among selected racial or ethnic populations during the 1950s-1960s and a projected percentage for the United States in 2025, with notes on potential demographic shifts over time [26,30].

Race or Ethnic Population	Time Period	LA %
Japanese	1950s–1960s	10.6%
Nigerians	1950s–1960s	9.2%
Bostonians	1950s–1960s	8.1%
Colombians	1950s–1960s	5.5%
Jamaicans	1950s–1960s	5.8%
United States	2025	21.5%

The diversity of fatty acid compositions in cooking oils significantly influences their nutritional impact, with LA serving as a polyunsaturated fatty acid (PUFA) that varies widely across oil types [31,32]. To show this variation, Table 2 presents the LA content in a range of cooking oils, derived from established compositional analyses.

**Table 2:** Linoleic acid (LA) content in common cooking oils *“Vegetable oil” is a generic blend; LA content varies depending on the specific blend. LA content in common cooking oils, presented in grams per 100 grams.

Cooking Oil	LA (g per 100 g of cooking oil)
Vegetable oil*	Depends on specific oil
Safflower	74.6
Sunflower	65.7
Cottonseed	51.5
Corn	53.5
Soybean	50.3
Canola	18.6
Olive	9.8
Butter oil	2.3
Coconut	1.8

The proposition that dietary shifts, particularly the elevated consumption of omega-6 PUFAs, may drive increased cancer risk through mechanisms such as altered membrane phospholipid composition and heightened oxidative stress merits detailed examination [33-36]. This reviews looks at the specific pathways, including lipid peroxidation (oxidative degradation of membrane fats) and pro-inflammatory cytokine production (release of signaling molecules that promote chronic inflammation), alongside evidence linking omega-6 fatty acids, exemplified by LA, to carcinogenesis By situating these findings within the broader narrative of lifestyle-driven oncogenesis, this review aims to clarify how historical changes in dietary habits intersect with cellular mechanisms to influence cancer prevalence.

The modern diet furnishes LA in such abundance that deficiency is a theoretical rather than practical concern, despite the human body’s inability to synthesize it endogenously. For humans, an intake of just 2 grams per day suffices to avert deficiency symptoms. The current Western intake of approximately 18 grams daily far exceeds this [37-39]. Consequently, LA deficiency is implausible outside of extreme, fat-free regimens, such as total parental nutrition (TPN), highlighting a significant disconnect between its biochemical essentiality and its overwhelming presence in modern nutritional contexts.

Nutrients are traditionally categorized based on their endogenous synthesis capacity [40], with essential nutrients requiring dietary intake and conditionally essential nutrients necessitating supplementation during periods of increased demand or metabolic stress. That binary misses a third reality: some nutrients are biochemically essential yet consistently plentiful in modern diets, making deficiency near-impossible. This paper proposes calling this third group Ubiquitous Essential Nutrients (UENs), defined by four criteria listed in Table 3.

**Table 3:** Criteria defining ubiquitous essential nutrients (UEN) These four criteria define a distinct category of nutrients that, while biochemically essential, are so abundant in modern diets that deficiency is rare, and the primary concern shifts to the risks associated with chronic oversupply. This reframing of essentiality, as applied to nutrients such as linoleic acid, underscores the need to balance intake to optimize health outcomes rather than merely prevent deficiency.

Criterion	UEN Requirement
1	Humans lack the enzymes to synthesize them
2	Average intake is ≥ 5 fold above the minimal requirement
3	Deficiency appears only in artificial or pathological states
4	Chronic oversupply carries documented risk

Recognizing UENs shifts focus from deficiency prevention to managing upper limits. For LA, while 1-2% of energy suffices for growth [41], U.S. consumption nears 5-10%, with adipose LA doubling since the 1960s [26], raising concerns about oxidized metabolites and altered CL composition. This reframing could refine dietary guidelines, therapeutic formulations, and public health messaging.

LA: essential and irreplaceable in mitochondrial function

Notably, the absence of direct measurements of LA in cardiolipin (CL) among low-LA populations limits definitive conclusions [42]. CL, a dimeric phospholipid unique to mitochondria, is indispensable for ATP synthesis. In oxidative tissues like cardiac and skeletal muscle, tetralinoleoyl-cardiolipin (LA₄CL), comprising four LA chains, dominates, constituting 70-80% of total CL [43]. LA’s two double bonds confer flexibility to the inner mitochondrial membrane (IMM), enabling the formation of cristae [44,45], tightly folded structures that maximize surface area for oxidative phosphorylation [46]. This curvature supports the dense packing of respiratory chain Complexes I, III, and IV, enhancing electron flux through supercomplexes and promoting efficient mitochondrial respiration [47-49].

LA’s designation as an essential fatty acid (EFA) stems from the absence of Δ-6 and Δ-5 desaturase enzymes in mammals, necessitating dietary intake for cellular membrane integrity and eicosanoid biosynthesis, which regulates inflammation [50,51]. This classification originates from Burr and Burr’s 1929-1930 experiments [52], where rats consuming 0.6% of calories from LA showed improved growth and did not develop skin pathologies, unlike fat-deficient controls. In this context, the most critical biological role of LA lies not in these historically prioritized functions but in its indispensable contribution to CL synthesis, which underpins mitochondrial function [53].

**Figure 3:**
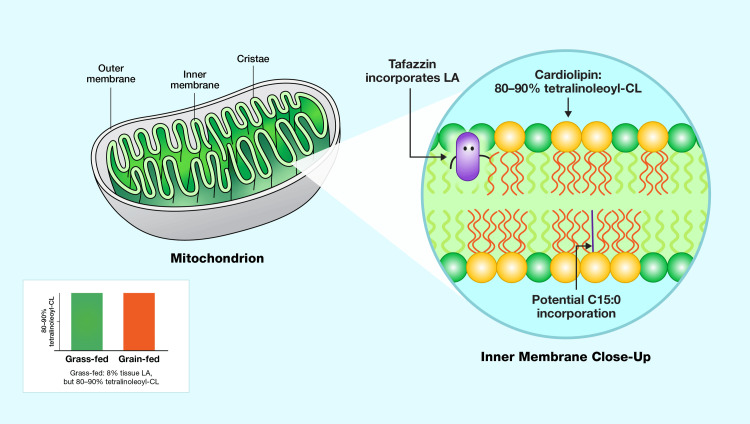
Role of linoleic acid in mitochondrial cardiolipin composition Role of  linoleic acid (LA) in mitochondrial cardiolipin (CL), highlighting its dominance in tetralinoleoyl-cardiolipin (LA₄CL) (80–90% of total CL) and the enzyme TAZ’s role in LA incorporation [41]. The inset compares CL composition in grass-fed and grain-fed cows, showing consistent LA dominance despite dietary differences [55,57]. The figure also hints at the potential for C15:0 substitution. Image Credit: Joseph Mercola, DO

To address this gap, studies of grass-fed cows, which exhibit tissue LA levels akin to those of indigenous humans (approximately 8%) [54], provide a valuable proxy. Remarkably, these cows maintain CL compositions with 80-90% LA₄CL, mirroring levels observed in grain-fed counterparts (Figure 3) [55]. This efficient incorporation is orchestrated by the enzyme tafazzin (TAZ), which prioritizes LA for CL synthesis and is essential for maintaining mitochondrial function [56]. This finding suggests that, even at reduced tissue LA concentrations, LA is efficiently incorporated into CL, ensuring sufficient membrane fluidity and mitochondrial ATP production.

TAZ facilitates this incorporation, preserving the same structural benefits [58]. This structural adaptation, while enhancing energy production, reflects an evolutionary trade-off, as LA’s unsaturation increases susceptibility to peroxidation [59]. In this context, Barth syndrome, a disorder arising from TAZ gene mutations, illustrates LA’s necessity [60]. Affected individuals exhibit a CL profile marked by a 50-70% reduction in total CL content and a pronounced depletion of LA₄CL [61].

Without functional TAZ, LA cannot be properly incorporated into CL, resulting in defective CL remodeling. This leads to abnormal cristae ultrastructure, which reduces the density of ETC complexes and impairs ATP synthesis [62]. Historically, this mitochondrial energy deficit was fatal in infants prior to the advent of modern medical interventions [63].

Inflammation sets cancer trajectory: acute surveillance vs chronic tumor promotion

While LA supports cellular energy production under normal conditions, its dysregulation highlights an important link to the immunosuppressive mechanisms that influence cancer initiation and progression. The intricate interplay between the immune system and metabolic processes significantly shapes cancer biology. Inflammation serves as a double-edged sword in the context of cancer [64,65].

Acute inflammation plays a highly protective role by recruiting cytotoxic immune cells, such as cytotoxic T lymphocytes (CTLs) and natural killer (NK) cells, to induce apoptosis in malignant cells [66,67]. This process involves a local surge in perforin and granzyme B, enabling CTLs to activate the caspase-mediated intrinsic pathway of cell death [68,69]. Historically, such acute inflammatory responses have been prioritized for immediate pathogen clearance and remain essential for immune surveillance.

Conversely, when inflammation becomes chronic, persistently elevated interleukin‑6 (IL‑6) levels activate nuclear factor kappa B (NF-κB) [70], creating a tumor‑supportive microenvironment [71]. The activation of NF-κB upregulates anti-apoptotic proteins, such as B-cell lymphoma 2 (Bcl-2), allowing malignant cells to evade programmed cell death. Over time, this chronic inflammatory environment promotes genetic instability, enhances proliferative signaling, and confers resistance to apoptosis [72].

**Figure 4:**
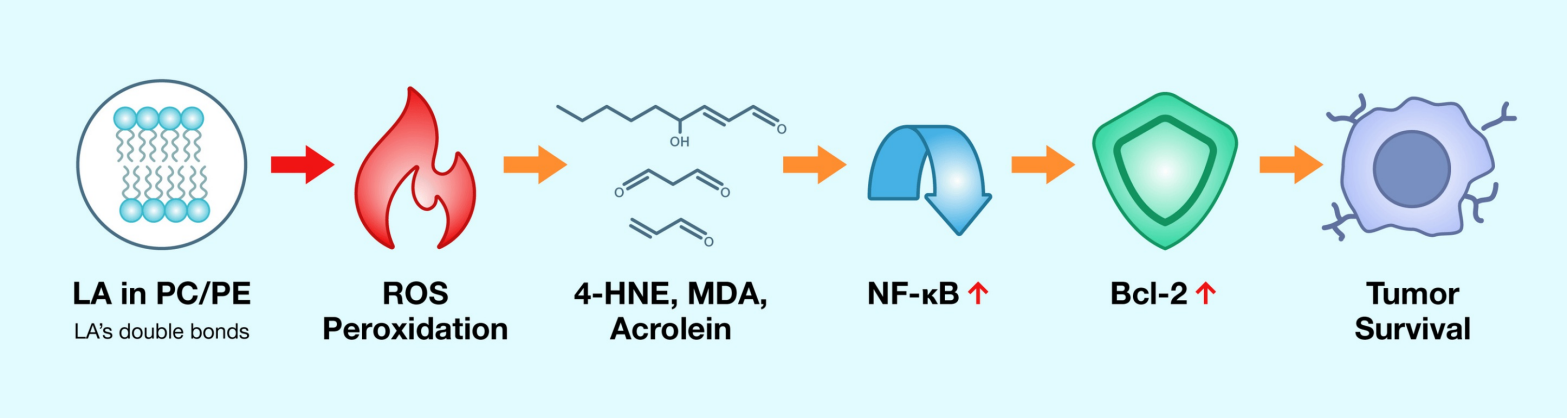
Molecular pathway from LA peroxidation to tumor survival This flowchart details the steps from LA in membrane phospholipids to lipid peroxidation, generation of toxic aldehydes (e.g., 4-HNE, MDA), activation of NF-κB, upregulation of Bcl-2, and ultimately, tumor cell survival. LA: linoleic acid; PC: phosphatidylcholine; PE: phosphatidylethanolamine; ROS: reactive oxygen species Image Credit: Joseph Mercola, DO

Building on this cytokine‑driven NF‑κB activation, excess LA in cell membranes increases lipid peroxidation in tissues, generating reactive aldehydes such as 4-hydroxynonenal (4-HNE), malondialdehyde (MDA), and acrolein [73,74]. These electrophilic byproducts act as “toxic second messengers” that propagate oxidative stress and modulate cellular signaling. The biochemical microenvironment shaped by persistent LA-derived aldehydes differs from an acute inflammatory response [75]. Acute inflammation, with its robust and transient cytokine bursts, often promotes cancer cell apoptosis; in contrast, chronic inflammation fueled by ongoing oxidative damage paradoxically enables tumor cell survival [76]. To synthesize these mechanistic steps into a unified framework, Figure 4 illustrates the complete cascade from dietary LA to tumor survival, providing a visual reference for the pathway discussed above.

Immunosuppression in cancer: tumor strategies and dietary influences

Chronic inflammation and immunosuppression interact in a deleterious feedback loop that promotes cancer progression [77,78]. Persistent inflammation, driven by the production of tumor necrosis factor alpha (TNF-α) from aldehydes derived from LA peroxidation, activates NF-κB [79], which in turn fosters an immunosuppressive microenvironment. This immunosuppression involves the upregulation of programmed death-ligand 1 (PD-L1) [80] on tumor cells, along with the recruitment of regulatory T cells (Tregs) [81] and myeloid-derived suppressor cells (MDSCs) [82,83], significantly dampening the function of CTLs and NK cells [84]. Consequently, this immunosuppressive state impairs the extrinsic apoptotic pathway, particularly when Fas (CD95) ligation is downregulated on tumor cells, allowing cancer cells to evade immune-mediated destruction [85-86]. Consequently, a biochemical cycle characterized by elevated oxidative stress, immunosuppression, and anti-apoptotic signaling sustains tumor survival and progression.

Concurrently, the peroxidation products of cellular membrane LA contribute to immune dysregulation [87]. These reactive aldehydes directly inhibit immune cell proliferation and function. Additionally, acrolein impairs macrophage phagocytosis and diminishes the production of cytokines like interleukin-12 (IL-12), weakening innate immune defenses [88]. Historically, the increased consumption of LA-rich diets has been associated with a rise in immune-related disorders, emphasizing the role of metabolic factors in immune regulation [89].

In addition to its effects on the immune system, LA directly promotes oncogenic signaling within tumor cells through fatty acid-binding protein 5 (FABP5), a cytosolic chaperone for hydrophobic molecules, converting it into an activator of oncogenic signaling. Specifically, LA binds to FABP5 with high affinity and increases its expression in neoplastic tissues. It promotes its interaction with Raptor, an important subunit of the mechanistic target of rapamycin complex 1 (mTORC1). This interaction enhances mTORC1 activity, thereby amplifying mitogenic signaling pathways that stimulate cellular proliferation [90].

In aggressive malignancies, such as triple-negative breast cancer, where FABP5 levels are markedly elevated, this protein correlates strongly with unfavorable clinical outcomes [91]. Experimental evidence demonstrates that genetic suppression of FABP5 attenuates tumor growth in preclinical models [92], whereas LA-bound FABP5 exacerbates it. High consumption of LA-rich vegetable oils potentiates the FABP5-mTORC1 axis, establishing a direct biochemical mechanism that links modern dietary patterns to an elevated risk of cancer [78]. Thus, high intake of LA not only contributes to an immunosuppressive tumor microenvironment but also directly fuels cancer cell proliferation [93], highlighting the multifaceted role of dietary fatty acids in cancer progression.

Why LA poses greater risk in the phospholipid bilayer than in adipose triacylglycerol

The phospholipid bilayer is primarily composed of phosphatidylcholine (PC) and phosphatidylethanolamine (PE) [94]. Upon excessive dietary intake of LA, it is preferentially esterified at the sn-2 position of these phospholipids [95]. In metabolically active tissues, such as skeletal muscle, LA constitutes approximately 15-20 mol % of membrane acyl chains, a level nearly threefold higher than the 18-23 mol % observed in circulating erythrocytes [96,97]. By contrast, its concentration in adipose triacylglycerol, where LA is stored in a relatively inert state, remains markedly lower, highlighting a significant disparity in its distribution.

**Figure 5:**
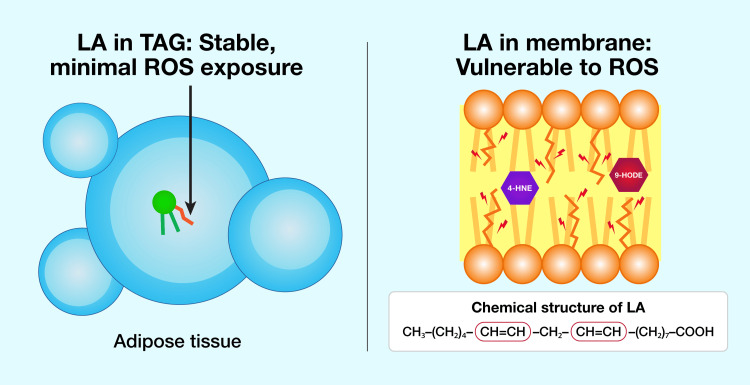
Differential peroxidation risk: adipose tissue vs. cell membranes Differential peroxidation risk of LA in adipose tissue versus cell membranes. In adipose tissue (left), LA is stored in triacylglycerol, shielded from ROS. In cell membranes (right), LA’s bis-allylic chains are exposed to ROS, leading to lipid peroxidation and the generation of toxic aldehydes such as 4-HNE. LA: linoleic acid; ROS: reactive oxygen species; TAG: triacylglycerol Image Credit: Joseph Mercola, DO

Positioned at the aqueous interface of the phospholipid bilayer, LA’s bis-allylic chains are incessantly exposed to reactive oxygen species (ROS) that are generated by mitochondria, peroxisomes, and nicotinamide adenine dinucleotide phosphate (NADPH) oxidase [98]. This oxidative environment starkly differs from the hydrophobic core of adipose triacylglycerol, where LA remains shielded from such reactive entities (Figure 5) [99]. Consequently, hydroperoxides accumulate on LA-containing PC (LA-PC) within minutes following an oxidative burst.

In contrast, under identical conditions, hydroperoxides on trilinolein, a triacylglycerol storage form of LA, exhibit negligible formation, underscoring the relative stability of LA in adipose tissue. This peroxidation process transforms LA-PC and LA-PE into a diverse array of oxidized phospholipids (OxPLs) and oxidized LA metabolites (OXLAMs), including 9-hydroxyoctadecadienoic acid (9-HODE), 13-hydroxyoctadecadienoic acid (13-HODE), epoxyoctadecenoic acids (EpOMEs), and dihydroxyoctadecenoic acids (DiHOMEs) [100]. These bioactive lipids act as damage-associated molecular patterns (DAMPs), triggering innate immune responses [101]. Simultaneously, OxPLs engage pattern-recognition receptors, such as CD36 and Toll-like receptor 4 (TLR4), sustaining NF-κB activation. This, in turn, upregulates anti-apoptotic proteins, including members of the Bcl-2 family and myeloid cell leukemia-1 (Mcl-1), establishing a persistent anti-apoptotic state [102,103].

In effect, LA within the phospholipid bilayer functions as a molecular trigger: its susceptibility to oxidation generates lipid electrophiles that chronically activate innate immune circuits and impair apoptotic pathways. Historically, the shift toward Western dietary patterns has elevated the omega-6 to omega-3 PUFA ratio beyond 15:1, doubling LA enrichment in human cellular membranes and correlating with the modern rise in inflammation-driven malignancies [104]. Conversely, LA sequestered within adipose triacylglycerol resides in a hypoxic, antioxidant-depleted core, largely insulated from ROS and incapable of initiating this deleterious cycle [105]. Thus, minimizing LA incorporation into the phospholipid bilayer, or aggressively neutralizing ROS at this interface, appears vital to interrupting the sequence of oxidative stress, inflammation, and apoptosis resistance that heightens cancer susceptibility. Understanding how LA contributes to membrane vulnerability raises an important question: how rapidly can dietary interventions alter membrane composition? The answer lies in the kinetics of phospholipid turnover.

Phospholipid turnover in cell membranes

Having established that the primary risk associated with LA stems from its incorporation into membrane phospholipids [106-108], where it is prone to peroxidation and the generation of pro-inflammatory oxidative metabolites, it is imperative to explore mechanisms for mitigating this risk. Central to this endeavor is a detailed understanding of phospholipid turnover, the dynamic process by which phospholipids are synthesized, modified, and degraded within cellular membranes [109,110].

Stable‑isotope pulse‑chase experiments show that about half of the phospholipids in a “typical” mammalian membrane are renewed every 48-72 hours (≈2-3 days) [111], yet these half‑lives can swing nearly an order of magnitude depending on the cell type and the membrane compartment being studied [112]. In erythrocytes, metabolically quiescent, anucleate cells, the dominant phospholipid, PC, is renewed roughly every 9-12 days, paralleling the cell’s 120‑day circulatory lifespan [113]. Whereas in metabolically active hepatocytes, the bulk pool is renewed in only ~24-48 hours [114,115], a tempo that supports high‐throughput lipid trafficking, detoxification, and lipoprotein secretion.

Even within a single cell, turnover is compartmentalized: endoplasmic‑reticulum and Golgi membranes, sites of active biosynthesis and vesicle budding, cycle their phospholipids markedly faster than the relatively stable plasma membrane. This dynamic equilibrium is orchestrated by coupling catabolic enzymes such as phospholipase A₂, which hydrolyses sn‑2 acyl chains [116], with anabolic counterparts like choline phosphotransferase that drive de novo PC synthesis [117]. Together, these processes balance membrane integrity with the rapid compositional flexibility required for signaling and adaptive stress responses [118]. The dynamic nature of membrane remodeling suggests that compositional changes achieved through dietary modification could manifest within days to weeks, a timeframe relevant for both prevention and therapeutic intervention.

Membrane lipids in therapy and prevention

The same phospholipid turnover processes that sustain membrane function also create targets for therapeutic and preventive intervention. While contemporary oncology deploys increasingly elaborate anti-inflammatory pharmacotherapies to address the transcription factors and cytokine signaling in chronic inflammation, these approaches do not address the root cause tied to membrane lipid profiles [119,120].

In this context, targeting the biochemical framework that triggers the inflammatory cascade proves significantly more efficacious than perpetually suppressing its downstream manifestations. An imbalanced lipid profile modifies membrane fluidity, impairs lipid-raft-mediated signaling, and predisposes toll-like and G-protein-coupled receptors to excessively activate the NFκB transcriptional complex [121], widely recognized as the molecular initiator of chronic inflammatory states [122]. Furthermore, empirical evidence demonstrates that replenishing the membrane lipid reservoir with anti-inflammatory constituents or their precursors effectively restores these signaling thresholds [123]. For example, enrichment with PC attenuates lipopolysaccharide-induced NFκB activation, reducing downstream IL-6 and TNF-α release, as observed in both in vitro cellular models and in vivo animal studies [124].

Consequently, therapeutic strategies designed to recalibrate membrane composition, such as dietary rebalancing of fatty acids and targeted supplementation with phospholipids, or modulation of endogenous desaturase enzyme activity, address the initial step in the pathogenic progression [125,126]. By contrast, pharmacological inhibitors of NFκB operate analogously to suppressants applied after an alert has been triggered: they diminish the inflammatory response temporarily but fail to rectify the underlying membrane dysregulation, thereby permitting recurrent inflammatory episodes upon cessation of treatment [127,128]. Interventions that normalize membrane lipid composition may yield more durable benefits than repeated pharmacologic blockade of downstream inflammatory mediators [129].

Since Virchow’s 1863 observation that chronic irritation can initiate neoplasia anticipated current recognition of cancer as, in part, an inflammation-driven disease [130]. Furthermore, chronic, low-grade inflammation demonstrably accelerates tumor progression and confers resistance to radio‑ and chemotherapy, underscoring the imperative to extinguish the initiating insult rather than perpetually damp its downstream cascades with synthetic chemistry [131,132].

Strategically reducing dietary LA intake along with targeted PC and PE supplementation can restore membrane phospholipid balance and short‑circuit the chronic inflammatory loop that fuels tumor initiation and progression [133]. Such strategies enhance immune function and alleviate chronic inflammation, thereby supporting immunotherapeutic interventions [77,134]. Historically, diets with lower LA ratios have been linked to improved outcomes in oxidative stress-related conditions, reinforcing the preventive value of dietary adjustments [135-137].

Pentadecanoic acid: from ruminant fat to EFA

Pentadecanoic acid (C15:0) is an odd-chain saturated fat (OCFA) that primarily originates from ruminant animals [138], where gut microbiota ferment dietary carbohydrates into propionate, a precursor for the biosynthesis of OCFAs such as C15:0 and heptadecanoic acid (C17:0) [139,140]. In contrast, non-ruminants, including humans, exhibit limited endogenous synthesis of OCFAs [141], with minimal production via α-oxidation of phytanic acid in specific metabolic disorders, thus necessitating dietary intake as the predominant source of C15:0 [142], a dependency that historically contributed to its marginalization in lipid research despite its emerging physiological significance.

Historically, the designation of “essential” fatty acids has been reserved for PUFAs like LA and alpha-linolenic acid (ALA), which humans cannot synthesize de novo due to the absence of Δ12 and Δ15 desaturases [143]. Emerging scientific evidence now challenges this classical framework, strongly supporting the classification of C15:0 as an EFA, given its indispensable physiological roles and limited endogenous synthesis.

C15:0 was historically regarded as a minor dietary component, overshadowed by more abundant even-chain fatty acids like palmitic (C16:0) and stearic (C18:0) acids [144-146]. C15:0 constitutes less than 1-3% of total fatty acids in typical Western diets, contributing to its initial obscurity [139,147]. The lipid bilayer of cellular membranes owes much of its functionality to its fluidity, which is governed by the fatty acid composition of its phospholipids. Saturated fatty acids (SFAs), such as C16:0 and C18:0, characterized by their lack of double bonds, promote tight packing within the bilayer, thereby reducing fluidity. In contrast, PUFAs, exemplified by LA, introduce kinks into their hydrocarbon chains that enhance fluidity by disrupting this orderly arrangement [148]. While LA’s role in increasing membrane fluidity is well-documented, its susceptibility to oxidation presents a significant drawback [149].

By comparison, even-chain SFAs like C16:0 and C18:0 offer remarkable resistance to oxidation, ensuring the long-term stability of the lipid bilayer [150]. Yet, this stability comes at the cost of rigidity, limiting membrane flexibility. Thus, while even-chain SFAs mitigate the oxidative risks posed by LA, their impact on fluidity highlights an ongoing challenge in balancing stability and functionality within cellular membranes [151]. Table 4 provides a comprehensive comparison to inform dietary and therapeutic strategies aimed at optimizing membrane composition.

**Table 4:** Comparative characteristics of dietary fatty acids and choline *Choline is not a fatty acid but is included for its role in membrane phospholipids. Comparative traits of LA, ALA, palmitic acid (C16:0), stearic acid (C18:0), pentadecanoic acid (C15:0), and choline. The table highlights differences in double bonds, peroxidation index, membrane fluidity effects, oxidative by-products, and dietary sources, supporting the manuscript’s focus on membrane composition and cancer risk. Image Credit: Joseph Mercola, DO

Compound	Double Bonds	Peroxidation Index	Fluidity Effect	By-products	Dietary Sources
Linoleic Acid (LA)	2	Moderate	Increases membrane fluidity	4‑HNE, MDA and Acrolein	Vegetable oils, Seeds and Nuts
Alpha‑Linolenic Acid (ALA)	3	High	Increases membrane fluidity	4‑HNE, etc.	Flaxseed, Chia seeds and Walnuts
Palmitic Acid (C16:0)	0	Low	Decreases membrane fluidity	None	Palm oil, Meat and Dairy
Stearic Acid (C18:0)	0	Low	Decreases membrane fluidity	None	Cocoa butter, Beef fat and Lard
Pentadecanoic Acid (C15:0)	0	Low	Enhances fluidity vs. Even‑chain SFA	None	Dairy fat and Ruminant meat
Choline*	N/A	N/A	N/A	N/A	Egg yolks and Meat

C15:0: a novel regulator of membrane fluidity and cellular function

Recently, attention has turned to OCFAs, which provide a compelling alternative to LA. Unlike their even-chain counterparts, OCFAs possess an uneven number of carbon atoms, a structural feature that disrupts tight packing within the lipid bilayer, thereby enhancing fluidity [152,153]. Specifically, because an odd-numbered chain like C15:0 terminates on the opposite side of the bilayer midpoint compared with its partner leaflet, the last methyl group ends up out-of-phase relative to neighboring even-chain fatty acids such as C16:0 or C18:0 [154].

This misalignment prevents the two leaflets from interdigitating perfectly, resulting in looser lateral packing and increased gauche conformations in the acyl chains. Consequently, the membrane exhibits properties akin to having a single cis-double bond, which enhances fluidity without the associated oxidation risks [155-157]. Bacterial studies further support this mechanism, showing that the loss of odd or branched-chain fatty acids leads to membrane rigidification and impaired growth at low temperatures [158]. C15:0 has also been associated with improved cellular structure in aging cells and oxidative stress conditions [159].

Systemically, C15:0 activates the AMP-activated protein kinase (AMPK) pathway and downregulates mTOR signaling, as evidenced by elevated AMPK phosphorylation in murine models [160]. These molecular shifts accelerate phospholipid remodeling, favoring shorter-chain and monounsaturated fatty acids (MUFAs), for instance, increasing oleic acid (C18:1) levels by up to 15%, thereby preserving membrane flexibility under metabolic stressors such as hypoxia or nutrient deprivation [161].

Furthermore, C15:0 competes with LA and its downstream omega-6 polyunsaturated derivatives for incorporation into membrane phospholipids, reducing the availability of substrates prone to ferroptotic lipid peroxidation. In vitro studies utilizing human cell lines demonstrate that this displacement results in a 40% decrease in iron-catalyzed ROS generation, significantly bolstering membrane integrity under oxidative stress [162]. Consequently, C15:0 serves as a protective lipid that mitigates ferroptosis, a programmed cell death pathway implicated in neurodegenerative and cardiovascular disorders.

In summary, the unique packing behavior of this OCFA, coupled with its capacity to induce adaptive lipid remodeling through AMPK activation and its protective role against ferroptosis, endows C15:0 with the rare ability to partially substitute for LA’s fluidizing function without compromising membrane stability. Ongoing studies are exploring C15:0’s potential therapeutic applications in metabolic and neurodegenerative disorders, given its protective effects against oxidative stress and ferroptosis.

Choline deficiency: a dietary trigger of membrane instability and cancer risk

Choline, a precursor to PC, a major cell and mitochondrial membrane phospholipid, is consistently under-consumed in modern diets [163]. The Adequate Intake (AI), set to prevent hepatocyte damage, is 550 mg/day for men and 425 mg/day for women [164]; however, NHANES data indicate average intakes of only 402 mg and 278 mg, respectively [165], with over 93% of U.S. adults falling below the AI [166]. This chronic shortfall, evident since the establishment of dietary guidelines in the early 2000s, restricts PC availability for remodeling cellular and CL membranes.

Adequate choline intake remains challenging due to the limited number of dietary sources rich in this essential nutrient. To meet the recommended daily intake, one would need to consume substantial quantities of choline-dense foods, such as approximately 450 grams (1 pound) of beef or four to five egg yolks daily [167]. However, these foods are not commonly consumed in such amounts in typical diets, and fortified options remain scarce, making it difficult for most individuals to achieve sufficient choline levels through dietary means alone [168].

Emerging mechanistic insights underscore the pivotal roles of PC and PE in cellular membrane integrity and function, with PC comprising approximately 50% of the cell membrane and PE constituting 20-30% [169], [170]. The recently characterized mitochondrial choline transporter, SLC25A48, facilitates choline uptake into the inner mitochondrial membrane, enabling the synthesis of PC, a phospholipid critical for membrane fluidity and cellular signaling. In cells deficient in this transporter, superoxide production increases by approximately 30%, while cellular proliferation decreases by 40%, resulting in an oxidative stress profile reminiscent of that observed in tumor bioenergetics [171].

This deficiency disrupts the synthesis of PC, altering the PC-to-PE ratio, which is essential for maintaining mitochondrial and cell membrane stability [172]. While choline’s historical role in neurotransmitter synthesis, particularly acetylcholine (Ach), has been well-documented [168], contemporary research underscores its exponentially greater significance in maintaining cell membrane integrity through the synthesis of PC [173]. Consequently, targeted choline repletion-achieved through dietary fortification, supplementation, or co-administration with C15:0-offers a practical approach to restore membrane homeostasis and counteract lipid-peroxidation-driven oncogenesis, particularly when paired with strategies to reduce LA intake [174,175].

Limitations

This narrative review synthesizes historical, epidemiological, and mechanistic evidence to propose a membrane-centric hypothesis linking dietary LA to cancer risk. However, several limitations warrant acknowledgment.

First, the reliance on food-disappearance statistics and historical records introduces inherent uncertainty, as these data reflect population-level estimates rather than individual consumption patterns. Second, while temporal correlations between LA intake and cancer incidence are compelling, correlation does not establish causation; multiple confounding variables have evolved concurrently. Third, the mechanistic evidence derives predominantly from in vitro and animal models, which may not fully recapitulate human physiology. This review did not employ formal risk-of-bias assessment for individual studies; evidence from randomized controlled trials, observational cohorts, and preclinical models was synthesized qualitatively to build a coherent mechanistic framework rather than to generate pooled effect estimates. Fourth, adipose tissue fatty acid composition reflects long-term dietary intake but may not precisely mirror the dynamic phospholipid composition of all cell membranes. Finally, prospective randomized controlled trials directly testing the cancer-preventive efficacy of LA restriction combined with C15:0 and choline supplementation in humans are currently lacking.

Recommendations

To address these gaps, several research priorities emerge. Intervention studies should quantify the dose-response relationship between dietary LA reduction and biomarkers of oxidative stress, inflammation, and membrane composition in high-risk populations. Concurrently, trials should assess optimal intakes of C15:0 and choline for membrane stabilization. Lipidomic profiling would clarify whether ancestral fatty acid ratios can be restored and whether such changes correlate with reduced pro-inflammatory cytokine production. Mechanistic studies should elucidate whether C15:0 directly competes with LA for incorporation into CL and other phospholipids.

Future directions

Long-term prospective cohort studies with rigorous dietary assessment are essential to determine whether individuals maintaining lower LA intake and higher C15:0/choline consumption exhibit reduced cancer incidence. Biomarker discovery efforts should identify signatures that predict response to dietary lipid interventions. Comparative effectiveness research should evaluate dietary lipid modulation alongside conventional pharmacologic anti-inflammatory agents. Finally, mechanistic work should investigate whether the proposed dietary strategy influences tumor microenvironment composition, cancer stem cell plasticity, and resistance to chemotherapy or immunotherapy. Definitive evaluation of this hypothesis will require targeted clinical trials, detailed mechanistic studies, and population-level implementation research.

## Conclusions

This review advances a novel hypothesis that attributes the modern escalation in cancer incidence to dietary shifts, most notably the increased intake of LA. The analysis demonstrates that excess LA, when incorporated into cellular membrane phospholipids, undergoes lipid peroxidation, producing reactive aldehydes such as 4-HNE. These electrophiles activate NF-κB, which upregulates anti-apoptotic proteins like Bcl-2, thereby promoting a pro-tumorigenic microenvironment characterized by chronic inflammation and oxidative stress. To address this, we propose a targeted dietary intervention utilizing C15:0 and choline. C15:0 may help stabilize cellular membranes and reduce oxidative stress by counteracting the effects of LA-derived aldehydes. In parallel, choline serves as a precursor to PC, enhancing membrane integrity and supporting lipid metabolism.

Historically, traditional diets with lower omega-6 content contrast sharply with contemporary patterns, underscoring the relevance of this approach. By restoring membrane homeostasis and mitigating inflammatory cascades, this strategy, which is rooted in evolutionary nutritional principles, offers a promising avenue for cancer risk reduction. Consequently, clinical trials are needed to validate the efficacy of C15:0 and choline supplementation, paving the way for evidence-based public health recommendations.
